# Inverse‐Designed Silicon Nitride Bimodal Mode Converter for Interferometric Sensors

**DOI:** 10.1002/nap2.70229

**Published:** 2026-07-25

**Authors:** Narges Dalvand, Julian L. Pita Ruiz, Boris Le Drogoff, Mohamed Chaker, Michaël Ménard

**Affiliations:** ^1^ Department of Electrical Engineering École de Technologie Supérieure (ÉTS) Montreal Quebec Canada; ^2^ Institut National de la Recherche Scientifique–Énergie, Matériaux Télécommunications (INRS‐EMT) Varennes Quebec Canada

## Abstract

High fringe visibility is essential for achieving high sensitivity and low detection limits in bimodal interferometric sensors, as it depends critically on balanced optical power between the interfering modes. Compact bimodal mode converters (BiMCs) that enable precise modal power control are therefore key components for integrated interferometric biosensors. However, conventional approaches based on shifted junctions or periodic waveguide structures often lack flexibility and exhibit limited efficiency, particularly for higher‐order mode excitation. Here, we experimentally demonstrate compact inverse‐designed BiMCs on silicon nitride (SiN) capable of exciting multiple supported mode pairs within footprints as small as 224 μm^2^ while maintaining near‐balanced modal power distributions. Devices were implemented on two SiN platforms with core thicknesses of 400 and 300 nm, confirming the versatility and fabrication robustness of the inverse‐design approach. Experimental characterization shows average coupling efficiencies of 41.1% and 46.0% across the C band, with a near‐ideal modal power balance of 45.1%/45.0% for the ${\text{TE}}_{00}$–${\text{TE}}_{02}$ pair. Furthermore, a complete ${\text{TE}}_{00}$–${\text{TE}}_{03}$ bimodal interferometer incorporating a 15‐mm multimode waveguide achieved an extinction ratio of 20.6 dB, corresponding to a fringe visibility of ∼0.98, among the highest reported for SiN bimodal interferometers.

## Graphical abstract

Inverse‐designed bimodal mode converters are demonstrated on two silicon nitride platforms. The devices convert the fundamental mode into near‐balanced pairs of the fundamental and higher‐order modes up to the third order. They are compact and fabrication‐tolerant. Characterization of a bimodal interferometer incorporating these converters demonstrates high extinction ratio and visibility, confirming accurate modal splitting.

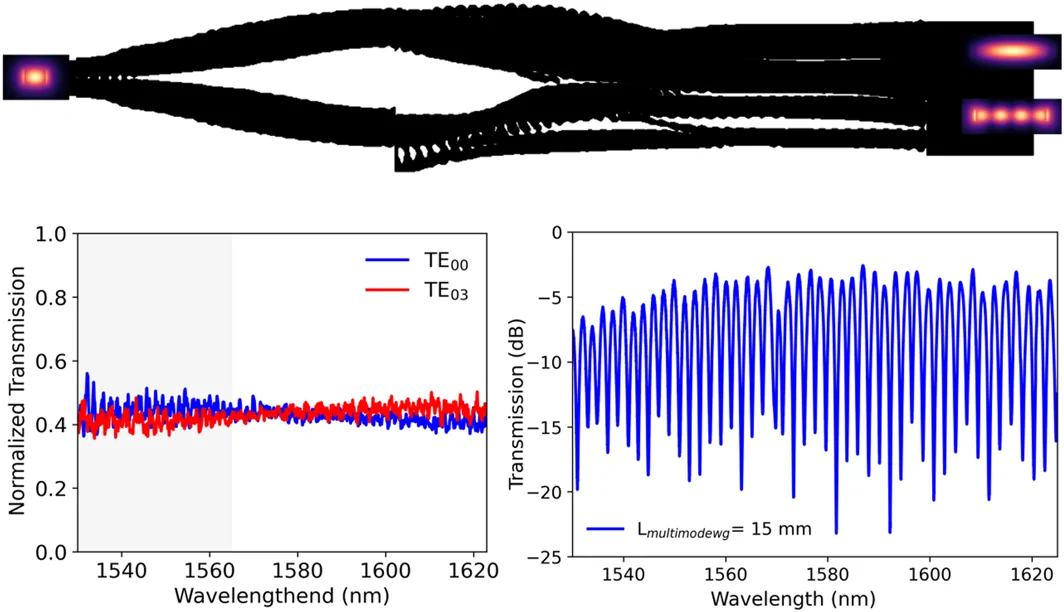

## 1 Introduction

Optical biosensors have attracted significant attention for point‐of‐care and real‐time diagnostic applications owing to their compactness, high sensitivity, and capability for label‐free detection [1, 2]. In many integrated sensing configurations, detection relies on the evanescent field of guided optical modes, where a fraction of the optical field extends beyond the waveguide core and interacts directly with the surrounding analyte. Variations in the refractive index of the cladding modify the effective index of the propagating mode, enabling rapid and direct optical detection without fluorescent labels or chemical markers [3, 4]. Such capabilities are particularly important for early stage disease diagnostics and biochemical monitoring applications [5, 6, 7, 8].

To enable compact and scalable sensing platforms, evanescent‐field biosensors are commonly implemented using integrated photonic technologies. Existing integrated sensing architectures include interferometric devices, such as Mach–Zehnder and Young interferometers [9, 10, 11, 12], resonator‐based structures including microring resonators and photonic crystal cavities [13, 14, 15, 16], as well as grating‐assisted sensing structures [17, 18]. Among these approaches, interferometric sensors generally provide the highest refractive‐index sensitivity, with Mach–Zehnder and Young interferometers capable of detecting refractive‐index variations on the order of $10^{-8}$ RIU [17]. However, conventional interferometric architectures typically require separate sensing and reference arms together with interaction lengths extending over several millimeters, leading to relatively large device footprints.

To reduce the footprint while preserving high sensitivity, bimodal waveguide (BiMW) interferometric sensors were proposed by Levy and Ruschin [19] and later experimentally demonstrated by Zinoviev et al. [20]. In these devices, interference occurs between two guided modes propagating within a single multimode waveguide, thereby eliminating the need for separate reference and sensing arms. A vertical thickness discontinuity introduced within a silicon nitride (SiN) waveguide enables simultaneous excitation of the fundamental and higher‐order modes [20]. Over the sensing region, the accumulated phase difference between the modes depends on the refractive index of the surrounding medium, generating an interference signal at the output. Although this configuration substantially reduces the sensor footprint, fabrication requires multiple lithography and etching steps to form the vertical discontinuity, increasing fabrication complexity.

In addition to compactness, achieving a low limit of detection (LOD) requires high interference visibility, which is directly determined by the optical power balance between the interfering modes. Consequently, precise control of the modal power distribution is essential for maximizing fringe visibility and improving sensing performance. This requirement has motivated increasing interest in compact and efficient bimodal mode converters (BiMCs).

Mode converters are widely used in integrated photonics, particularly in mode‐division multiplexing (MDM) systems for increasing communication capacity [21, 22]. Various mode‐conversion approaches have been explored, including multimode interference (MMI) couplers [23, 24], subwavelength Y‐junctions [25, 26, 27, 28], directional couplers [29, 30, 31], adiabatic tapers [32], spatial‐ and polarization‐division multiplexers [33], and inverse‐designed structures [34, 35, 36, 37, 38]. However, sensing‐oriented BiMCs have more complex requirements than conventional mode converters. Specifically, the device must simultaneously excite the fundamental and higher‐order modes from a single‐mode input while maintaining nearly balanced modal power distributions across a broad wavelength range. Efficient excitation of higher‐order modes under these constraints remains particularly challenging, especially within compact footprints on low‐index‐contrast platforms such as SiN.

Several strategies have been investigated to improve balanced bimodal excitation while preserving fabrication simplicity. In 2014, Liu et al. demonstrated a horizontal modal splitter based on spiral silicon waveguides to eliminate the need for multilayer fabrication processes [39]. Although the device achieved an interference contrast of approximately 15 dB, the spiral multimode waveguide introduced significant bending losses, limiting the reported sensitivity to 461.6 πrad/RIU. Torrijos‐Morán et al. later demonstrated subwavelength‐grating (SWG)‐assisted mode excitation on silicon [40], achieving a sensitivity of 2270 nm/RIU with a relatively short sensing length.

More recently, Fernández de Cabo et al. demonstrated a SWG‐based mode converter with customizable modal power ratios on a silicon‐on‐insulator (SOI) platform [41]. By employing graded‐index subwavelength segments, the device achieved a balanced ${\text{TE}}_{00}$/${\text{TE}}_{02}$ power distribution of approximately 45%/48% at 1550 nm. However, the periodic nature of SWG structures can introduce scattering losses and parasitic spectral oscillations associated with Fabry–Pérot‐like resonances, which may degrade fringe visibility and sensing accuracy. In addition, silicon platforms exhibit significant material absorption at visible wavelengths, limiting their suitability for many biosensing applications.

Polymer‐based bimodal sensors have also been investigated due to their fabrication simplicity and low cost. Liang et al. numerically demonstrated a long‐period‐grating‐assisted coupler exhibiting a near‐balanced 42%/43% power distribution and an extinction ratio of 43 dB [42]. In a subsequent experimental study, the same group demonstrated a lateral splitter based on an offset junction, achieving an extinction ratio of approximately 18 dB over a 5‐mm sensing length [8]. However, extending the sensing region to 10 mm resulted in a highly unbalanced modal distribution of approximately 77%/13% and a significantly reduced extinction ratio of 5.8 dB [43]. In general, polymer platforms suffer from higher propagation losses and limited thermal stability compared with SiN, restricting their compatibility with complementary metal–oxide–semiconductor (CMOS) fabrication processes [44, 45].

SiN has emerged as one of the most promising platforms for integrated biosensing due to its low propagation loss, CMOS compatibility, and broad transparency window extending from the visible to the near‐infrared. Nevertheless, achieving efficient and balanced excitation of higher‐order modes within compact SiN structures remains challenging because of the relatively low refractive‐index contrast of the platform. Torres‐Cubillo et al. demonstrated a SiN bimodal sensor based on coherent phase readout with a sensitivity of 4284 rad/RIU [46]. However, the device required a combined MMI, phase‐shifter, and Y‐branch configuration with a total length of approximately 290 μm. Laterally shifted junctions have also been proposed for compact SiN bimodal excitation [47], but these structures primarily excite only the first higher‐order mode and exhibit nonnegligible excitation of undesired modes.

Isayama and Hernández‐Figueroa et al. investigated directional couplers for the excitation of higher‐order modes on SiN platforms, numerically demonstrating balanced excitation of the ${\text{TE}}_{00}$ (47.9%) and ${\text{TE}}_{04}$ (47.9%) modes with a predicted LOD of $1.9\times 10^{-7}$ RIU [5]. In a subsequent work, the same group proposed a sensor based on interference between the fundamental TE and TM modes [6]. Although simulations predicted high sensitivity, practical implementation remains challenging because the polarization manipulation required for the excitation of the TM‐mode often introduces substantial insertion losses and experimental complexity. Other SiN implementations based on periodic structures within the sensing region have also been explored [48, 49], but these approaches similarly suffer from increased scattering losses and parasitic spectral oscillations.

Inverse design has recently emerged as a powerful strategy for realizing ultra‐compact photonic components with functionalities that are difficult to achieve using conventional design methodologies. Significant progress has been reported across several material platforms, including tellurite [50], diamond [51], lithium niobate [52], and silicon nitride [34, 35, 36, 53, 54, 55, 56]. Despite these advances, the realization of fabrication‐robust inverse‐designed BiMCs capable of balanced excitation of selected higher‐order modes on SiN platforms remains largely unexplored.

In this work, we experimentally demonstrate four compact inverse‐designed BiMCs implemented on two SiN platforms with core thicknesses of 400 and 300 nm. On the 400‐nm platform, devices targeting the ${\text{TE}}_{00}$–${\text{TE}}_{01}$, ${\text{TE}}_{00}$–${\text{TE}}_{02}$, and ${\text{TE}}_{00}$–${\text{TE}}_{03}$ mode pairs are demonstrated, whereas on the 300‐nm platform, the ${\text{TE}}_{00}$–${\text{TE}}_{03}$ mode pair is investigated. The proposed devices achieve balanced modal excitation within footprints as small as 26 μm × 7 μm for the ${\text{TE}}_{00}$–${\text{TE}}_{01}$ device and 32 μm × 7 μm for the higher‐order mode pairs. Experimental measurements demonstrate average modal transmission efficiencies ranging from 41.1% to 46.0% across the C band while suppressing undesired modes below 2.4%. Furthermore, a complete interferometer based on the ${\text{TE}}_{00}$ and ${\text{TE}}_{03}$ modes is experimentally demonstrated using a 15‐mm multimode waveguide, achieving an extinction ratio of 20.6 dB, corresponding to a fringe visibility of approximately 0.98. These results establish inverse‐designed SiN BiMCs as compact, fabrication‐robust, and highly versatile building blocks for next‐generation integrated interferometric platforms aimed at future biosensing applications.

## 2 Device Design and Methods

The proposed BiMCs are designed to transform the fundamental ${\text{TE}}_{00}$ mode supported by a single‐mode input waveguide into two co‐propagating modes within a multimode output waveguide: the fundamental mode and a selected higher‐order mode (${\text{TE}}_{01}$, ${\text{TE}}_{02}$, or ${\text{TE}}_{03}$). The primary design objective is to achieve a near‐balanced 50%/50% power distribution between the two target modes in order to maximize interferometric fringe visibility, which is expected to contribute to improved LOD in BiMW sensors.

The inverse‐design framework consists of three main sections: a single‐mode input waveguide, a free‐form optimization region, and a multimode output waveguide, as illustrated in Figure 1a. Although the multimode output waveguide supports up to four guided TE modes, the optimization was restricted to two target modes: the ${\text{TE}}_{00}$ mode and one selected higher‐order mode. All devices were optimized for the TE polarization over the C band using the adjoint‐based topology optimization framework implemented through the Python‐based LumOpt package from Ansys Lumerical. A minimum feature size (MFS) constraint of 120 nm was imposed during optimization. The figure of merit (FOM) was defined to maximize the transmission into the two target modes while simultaneously suppressing undesired modes, as detailed in Supporting Information S1: Note 1. Additional information regarding the topology‐optimization formulation can be found in ref. [35].

**FIGURE 1:**
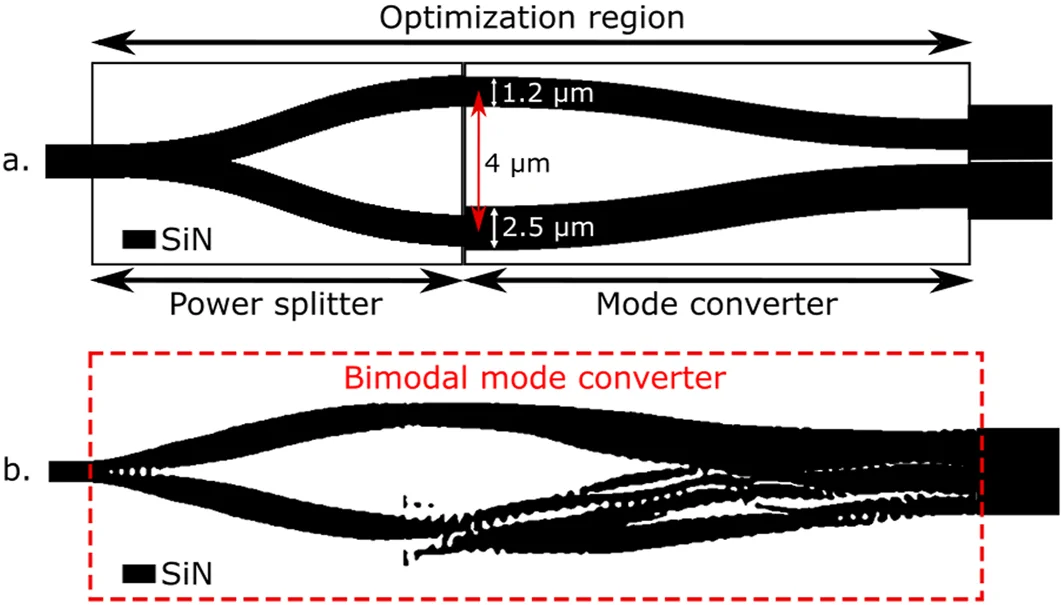
Design of the proposed BiMC. (a) Schematic of the inverse‐design framework and initial conditions. The optimization region is divided into a beam‐splitting section and a mode‐conversion section initialized using two SiN S‐bend waveguides. (b) Final optimized geometry for the ${\text{TE}}_{00}$–${\text{TE}}_{02}$ device, combining the beam splitter and mode‐conversion sections to form the complete BiMC.

Initial optimization attempts using an homogeneous ${\text{SiO}}_{2}$ or SiN refractive‐index distribution within the full optimization region failed to converge toward efficient solutions (Supporting Information S1: Figure S1). This behavior is primarily attributed to the complexity of simultaneously achieving beam splitting and higher‐order mode excitation within a single optimization domain. To improve convergence stability and transmission efficiency, the optimization process was separated into two functional stages.

As shown in Figure 1a, the design region was divided into a beam‐splitting section and a mode‐conversion section. The beam splitter first divides the optical power equally into two branches positioned symmetrically around the center axis with a separation of 2 μm. These two branches subsequently feed the mode‐conversion region, where the optical fields are spatially redistributed to selectively excite the target mode pair within the multimode output waveguide. Two SiN S‐bend waveguides were used to initialize the optimization geometry and improve convergence toward physically realizable solutions.

The alignment between the multimode output waveguide and the center of the mode‐conversion region was found to strongly influence the mode‐conversion efficiency. Introducing a lateral offset in the output waveguide improved the modal overlap and increased the conversion efficiency by approximately 5%. The optimal lateral offsets were 150 nm for the ${\text{TE}}_{00}$–${\text{TE}}_{01}$ device and 400 nm for the ${\text{TE}}_{00}$–${\text{TE}}_{03}$ device, whereas no offset was required for the ${\text{TE}}_{00}$–${\text{TE}}_{02}$ configuration.

Figure 1b presents the final optimized geometry for the ${\text{TE}}_{00}$–${\text{TE}}_{02}$ BiMC. Although an MFS constraint was included during optimization, a few sub‐MFS features remain visible in the final geometry, indicating that the constraint was not strictly enforced during all optimization stages.

The 50/50 beam splitter occupies a footprint of 7 μm × 12 μm. The complete ${\text{TE}}_{00}$–${\text{TE}}_{01}$ BiMC occupies a total design region of 7 μm × 14 μm, whereas the higher‐order ${\text{TE}}_{00}$–${\text{TE}}_{02}$ and ${\text{TE}}_{00}$–${\text{TE}}_{03}$ devices require a slightly larger footprint of 7 μm × 20 μm. Simulations were performed for SiN core thicknesses of 400 and 300 nm using the same inverse‐design methodology, whereas only the waveguide widths were modified to account for differences in modal confinement.

For the 400‐nm platform, three BiMCs targeting the ${\text{TE}}_{00}$–${\text{TE}}_{01}$, ${\text{TE}}_{00}$–${\text{TE}}_{02}$, and ${\text{TE}}_{00}$–${\text{TE}}_{03}$ mode pairs were designed. The single‐mode input and multimode output waveguide widths were 0.7 and 3 μm, respectively. Figure 2a presents the simulated electric‐field distributions at 1550 nm obtained using three‐dimensional finite‐difference time‐domain (3D FDTD) simulations. The first column shows the beam‐splitting section used in all BiMCs and its associated field profile. The remaining columns illustrate the selective excitation and propagation of the target modes for each BiMC configuration. Efficient spatial mode conversion are observed for all devices. Figure 2b shows the simulated transmission spectra from 1530 to 1625 nm. The shaded gray region denotes the C band used during optimization, whereas the insets provide magnified views of the undesired‐mode transmission. Across the C band, the average transmission efficiencies are 46.0% (${\text{TE}}_{00}$) and 46.4% (${\text{TE}}_{01}$) for the ${\text{TE}}_{00}$–${\text{TE}}_{01}$ device, 44.5% for both modes in the ${\text{TE}}_{00}$–${\text{TE}}_{02}$ device, and 45.1% for both modes in the ${\text{TE}}_{00}$–${\text{TE}}_{03}$ device. In the L band, the corresponding average transmissions are 49.1%/42.0%, 43.8%/45.4%, and 45.1%/42.7%, respectively.

**FIGURE 2:**
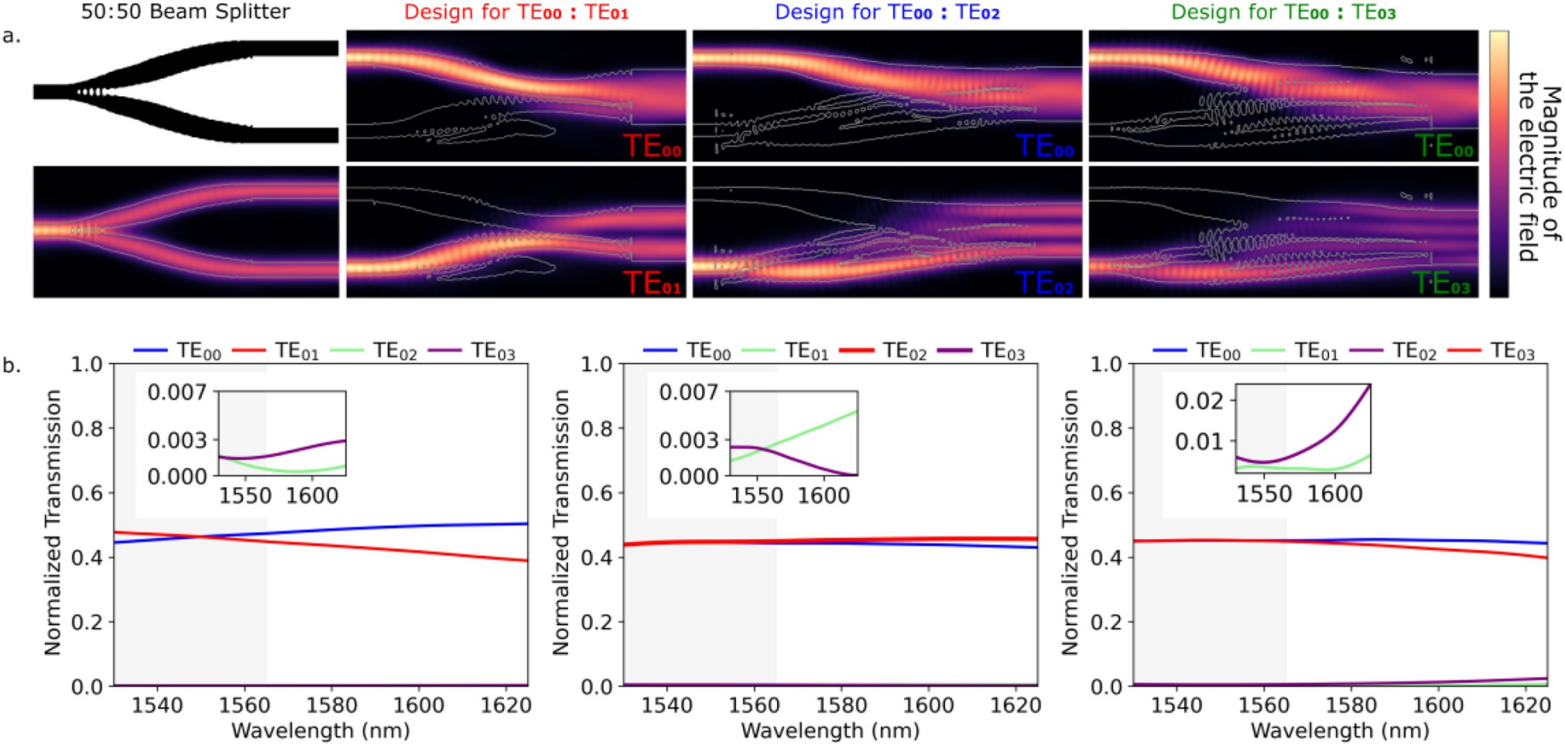
Simulation results for the inverse‐designed BiMCs on the 400‐nm SiN platform. (a) Electric‐field distributions obtained from three‐dimensional finite‐difference time‐domain (3D FDTD) simulations at 1550 nm. The first column presents the 50/50 beam splitter and its corresponding field profile. The remaining columns show the selective excitation and propagation of the ${\text{TE}}_{00}$–${\text{TE}}_{01}$, ${\text{TE}}_{00}$–${\text{TE}}_{02}$, and ${\text{TE}}_{00}$–${\text{TE}}_{03}$ mode pairs. (b) Simulated transmission spectra over the 1530–1625 nm wavelength range, including modal crosstalk. Insets provide a magnified view of the undesired‐mode transmission. The shaded region indicates the C band, which was used during the optimization.

Among the three devices, the ${\text{TE}}_{00}$–${\text{TE}}_{01}$ BiMC exhibits the largest wavelength‐dependent variation in the power‐splitting ratio across the C band, with a best‐case balanced split of 46.3%/46.3% at 1549.8 nm and a worst‐case split of 44.5%/47.6% at 1530 nm. In contrast, the ${\text{TE}}_{00}$–${\text{TE}}_{02}$ and ${\text{TE}}_{00}$–${\text{TE}}_{03}$ devices maintain highly uniform modal power distributions over the full operating wavelength range. For the ${\text{TE}}_{00}$–${\text{TE}}_{02}$ case, the splitting ratio remains above 44.4%/44.9% at the C band and above 43.0%/45.6% at the L band. Similarly, for the ${\text{TE}}_{00}$–${\text{TE}}_{03}$ case, the splitting ratio remains above 45.1%/44.9% at the C band and above 44.3%/39.7% at the L band. Undesired modes remain suppressed below 2.4%, confirming strong mode selectivity and low modal crosstalk.

To further evaluate the flexibility and fabrication robustness of the proposed inverse‐design methodology, a second SiN platform with a core thickness of 300 nm was investigated. The ${\text{TE}}_{00}$–${\text{TE}}_{03}$ mode pair was selected because efficient excitation of higher‐order modes while maintaining balanced power splitting is substantially more challenging than for lower‐order modes. In addition, higher‐order modes exhibit larger evanescent fields, which are advantageous for interferometric sensing applications.

For the 300‐nm platform, the widths of the single‐mode and multimode waveguides were increased to 1.3 and 5 μm, respectively, to accommodate the modified modal confinement conditions. Figure 3a presents the simulated electric‐field distributions for the ${\text{TE}}_{00}$–${\text{TE}}_{03}$ BiMC, demonstrating efficient selective excitation of the target modes. Figure 3b presents the corresponding simulated transmission spectra. Within the C band, the average transmission efficiencies are 48.3% for the ${\text{TE}}_{00}$ mode and 46.5% for the ${\text{TE}}_{03}$ mode. The ${\text{TE}}_{00}$ transmission remains highly stable between 48.3% and 49.0%, whereas the ${\text{TE}}_{03}$ transmission varies only between 46.0% and 46.6%. The best balanced split is obtained at 1530 nm, corresponding to 47.7%/46.6%, whereas the worst‐case split occurs at 1565 nm, with values of 48.8%/46.0%. Although a slight degradation in transmission is observed toward the L band, the device maintains a strong mode power balance. The average L band transmission is 49.0% for the ${\text{TE}}_{00}$ mode and 44.0% for the ${\text{TE}}_{03}$ mode, whereas the undesired ${\text{TE}}_{01}$ and ${\text{TE}}_{02}$ modes remain suppressed below 1.6%, confirming low modal crosstalk and stable balanced excitation.

**FIGURE 3:**
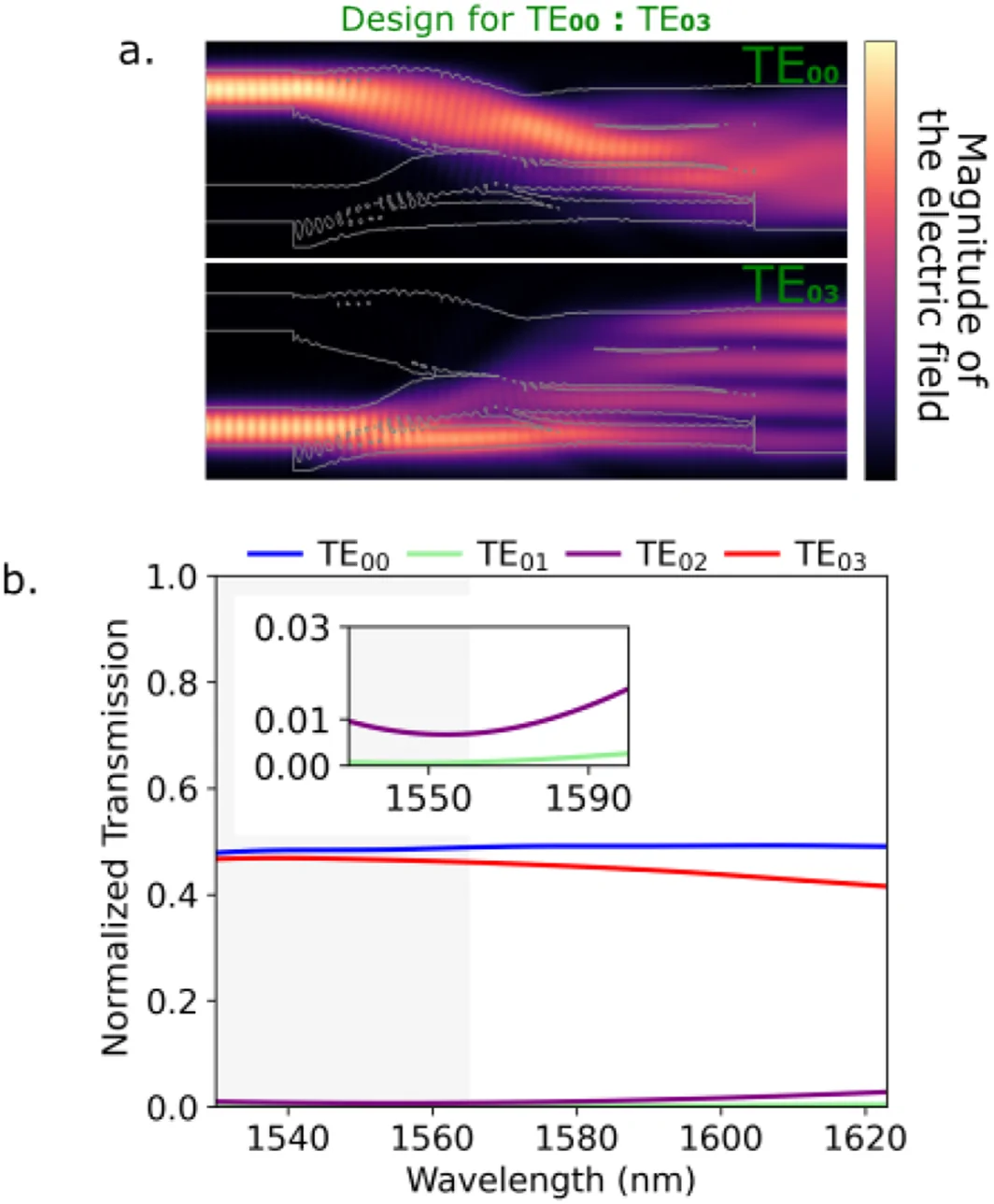
Simulation results for the inverse‐designed BiMC on the 300‐nm SiN platform. (a) Electric‐field distributions obtained from 3D FDTD simulations for the ${\text{TE}}_{00}$–${\text{TE}}_{03}$ mode pair. (b) Simulated transmission spectra over the 1530–1625 nm wavelength range together with modal crosstalk. Insets provide magnified views of the undesired‐mode transmission. The shaded region indicates the optimized C band.

To evaluate fabrication robustness, lateral dimensional variations of ± 10 and ± 20 nm were applied to all devices (Supporting Information S1: Note 2). Table 1 summarizes the average modal transmission across the C + L bands under these perturbations. Among the investigated devices, the ${\text{TE}}_{00}$–${\text{TE}}_{01}$ BiMC exhibits the highest sensitivity to fabrication variations. In particular, a 20‐nm lateral undersizing degrades the power balance to 55.6%/35.7% at 1565 nm. In contrast, the ${\text{TE}}_{00}$–${\text{TE}}_{03}$ devices implemented on both the 300 and 400 nm platforms demonstrate significantly improved fabrication tolerance. The largest degradation occurs under 20 nm lateral oversizing, where the power distributions at 1550 nm become 46.3%/36.7% for the 400 nm platform and 42.5%/36.0% for the 300‐nm platform. Despite these perturbations, the devices maintain relatively balanced modal excitation.

**TABLE 1:** Average transmission for each target mode pair across the C + L bands under nominal conditions (Nom) and under lateral undersizing and oversizing variations of 10 and 20 nm.

	Nom	Lateral undersizing (%/%)		Lateral oversizing (%/%)		Thickness
	(%/%)	10 nm	20 nm	10 nm	20 nm	(nm)
${\text{TE}}_{00}$/${\text{TE}}_{01}$	47.9/43.6	48.5/43.5	55.4/35.8	45.3/46.1	44.5/44.9	400
${\text{TE}}_{00}$/${\text{TE}}_{02}$	44.1/45.1	47.3/43.5	49.3/39.5	41.7/48.3	36.8/50.6	400
${\text{TE}}_{00}$/${\text{TE}}_{03}$	45.1/43.6	44.8/45.2	42.4/42.0	44.8/42.5	45.0/36.4	400
${\text{TE}}_{00}$/${\text{TE}}_{03}$	48.8/45.0	48.2/43.6	46.7/40.5	47.1/44.6	42.9/37.0	300

*Note:* Values are expressed as modal power distributions and rounded to one decimal place.

To further mitigate fabrication‐induced deviations on the 400 nm platform, a deep‐learning‐assisted correction model developed by PreFab Photonics [57] and trained on the Applied Nanotools Inc. (ANT) fabrication process was applied (see Supporting Information S1: Note 3).

The inverse‐design optimization was performed on a workstation equipped with an Intel Core i7‐9700 processor and 64 GB of RAM. The computational cost varied depending on device complexity. The BiMC optimizations required up to 152 iterations, with individual iteration times ranging from approximately 15 min to 1 h 40 min. In contrast, the beam‐splitter optimizations required up to 125 iterations with average iteration times of approximately 1 min.

Devices were fabricated using two independent SiN fabrication processes. For the 400‐nm platform, fabrication was performed at ANT through a multi‐project wafer run organized by CMC Microsystems. The substrate consisted of a 525‐μm silicon wafer with a 4.5‐μm buried oxide layer and a 3‐μm PECVD silica top cladding. Device patterns were defined in hydrogen silsesquioxane (HSQ) resist using 100 keV electron‐beam lithography (EBL).

For the 300‐nm platform, fabrication was carried out at the Laboratory of Micro and Nanofabrication of the Institut national de la recherche scientifique (INRS). The SiN layer was deposited by low‐pressure chemical vapor deposition (LPCVD) on a 3.5‐μm thermal oxide layer (WaferPro). Device patterns were defined in maN resist using 100 keV EBL and transferred into the SiN layer using inductively coupled plasma (ICP) etching. A 3.5‐μm PECVD silica top cladding was subsequently deposited.

Optical characterization was performed using an EXFO automated multi‐die testing station providing high alignment precision and repeatability. An EXFO T100S‐HP tunable laser and an EXFO CTP10 detector were used as the optical source and measurement system, respectively. Light was coupled into the devices using polarization‐maintaining (PM) 30° fiber arrays and grating couplers optimized for the TE polarization.

An asymmetric directional coupler (ADC) was used to separate the fundamental and higher‐order modes, as illustrated in Figure 4. The higher‐order mode was transferred into a single‐mode waveguide connected to an output grating coupler, whereas the fundamental mode remained within the multimode waveguide and was routed through a long adiabatic taper before coupling to a separate grating coupler. The simulated ADC insertion loss varied from 0.1 to 0.9 dB depending on the target mode. To accurately isolate the BiMC performance, grating‐coupler losses were removed using two loopback reference structures matched to the propagation lengths of the measured modes. ADC insertion losses were independently characterized using two ADCs connected back‐to‐back. Experimentally measured ADC losses ranged from 0.9 dB for the ${\text{TE}}_{01}$ mode to 2.1 dB for the ${\text{TE}}_{03}$ mode.

**FIGURE 4:**
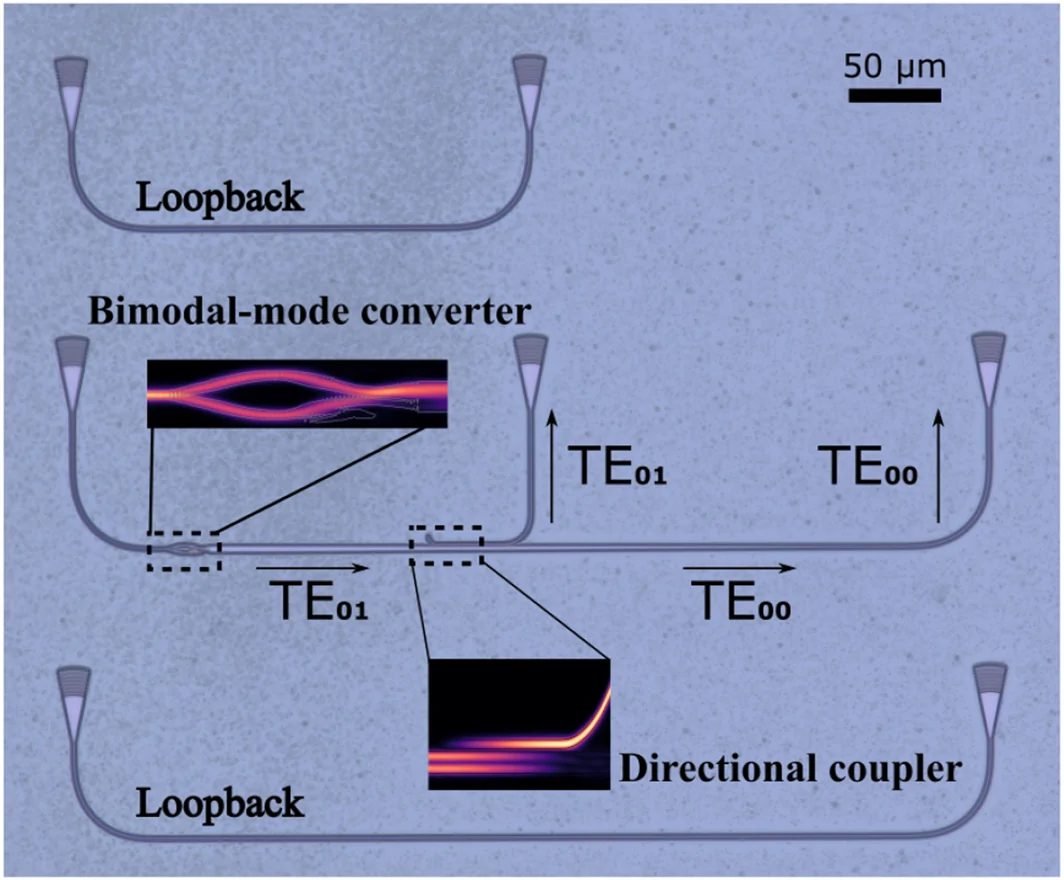
Schematic of the characterization circuit used for BiMC measurements. The BiMC is connected to an asymmetric directional coupler (ADC) used to separate the higher‐order and fundamental modes. Two loopback structures are included to remove the effects of propagation and grating‐coupler losses during normalization.

## 3 Results

Figure 5a presents the optimized geometries of the inverse‐designed BiMCs targeting the ${\text{TE}}_{00}$–${\text{TE}}_{01}$, ${\text{TE}}_{00}$–${\text{TE}}_{02}$, and ${\text{TE}}_{00}$–${\text{TE}}_{03}$ mode pairs on the 400‐nm SiN platform. The black regions correspond to the SiN core embedded within the ${\text{SiO}}_{2}$ cladding. The corresponding SEM images shown in Figure 5b confirm the high fabrication fidelity achieved using the ANT process. In particular, several sub‐MFS features are accurately reproduced despite being below the nominal 120‐nm minimum feature size. Features as small as approximately 90 nm, highlighted using colored circles in both the layouts and SEM images, remain clearly resolved after fabrication.

**FIGURE 5:**
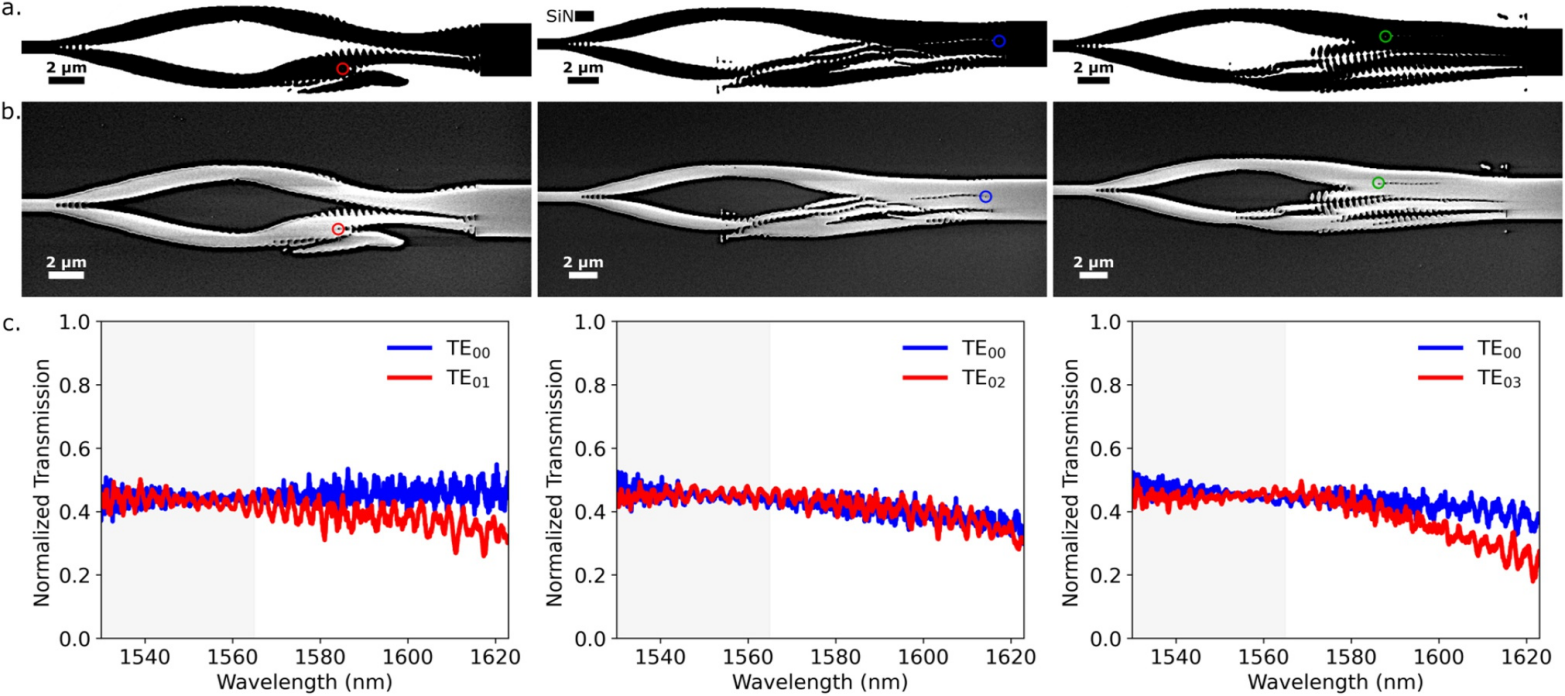
Fabricated inverse‐designed BiMCs and experimental characterization on the 400‐nm SiN platform. (a) Optimized geometries of the ${\text{TE}}_{00}$–${\text{TE}}_{01}$ (footprint: 26 µm × 7 μm), ${\text{TE}}_{00}$–${\text{TE}}_{02}$, and ${\text{TE}}_{00}$–${\text{TE}}_{03}$ (footprints: 32 μm × 7 μm) BiMCs. (b) SEM images of the fabricated devices obtained using the ANT fabrication process. Colored circles identify corresponding sub‐MFS features in both the layouts and SEM images. (c) Measured transmission spectra of the three BiMCs across the C and L bands. The color coding matches the corresponding mode pairs shown in Figure 2.

Figure 5c presents the experimentally measured transmission spectra across the C and L bands for the 400‐nm SiN devices. Within the C band, the average measured modal transmissions are 44.1%/43.5% for the ${\text{TE}}_{00}$–${\text{TE}}_{01}$ device, 45.1%/45.0% for the ${\text{TE}}_{00}$–${\text{TE}}_{02}$ device, and 46.0%/44.6% for the ${\text{TE}}_{00}$–${\text{TE}}_{03}$ device. Among the three configurations, the ${\text{TE}}_{00}$–${\text{TE}}_{02}$ BiMC exhibits the most balanced power distribution over the full measured wavelength range. At 1550 nm, the device achieves a power distribution of 45.5%/45.4%, whereas at 1625 nm, the transmission decreases to 29.8%/30.1%. Although the absolute transmission gradually decreases toward longer wavelengths, the relative modal power balance remains highly stable. At 1550 nm, the ${\text{TE}}_{00}$–${\text{TE}}_{01}$ device exhibits a modal power distribution of 42.1%/45.3%, whereas the ${\text{TE}}_{00}$–${\text{TE}}_{03}$ device achieves 45.7%/44.2%. The worst‐case splitting ratio for the ${\text{TE}}_{00}$–${\text{TE}}_{01}$ device occurs at 1617.23 nm, corresponding to 51.9%/27.3%. For the ${\text{TE}}_{00}$–${\text{TE}}_{03}$ device, the worst‐case split is measured at 1621.57 nm, corresponding to 35.8%/18.3%.

The spectral oscillations observed in the experimental measurements are primarily attributed to the grating‐coupler normalization process together with residual Fabry–Pérot resonances induced by subwavelength features within the devices. The oscillation amplitude reaches approximately 11% in the C band and 15% in the L band. Despite these oscillations, the measured spectra closely follow the trends predicted by the simulations. Table 2 summarizes the average C + L band performance together with the corresponding simulated values.

**TABLE 2:** Average transmission of BiMCs with SiN core thicknesses of 300 and 400 nm over the C + L band: Comparison of simulated (sim) and experimental (exp) results.

	Sim	Exp	Thick
	(%/%)	(%/%)	(nm)
${\text{TE}}_{00}$/${\text{TE}}_{01}$	47.9/43.6	44.9/39.9	400
${\text{TE}}_{00}$/${\text{TE}}_{02}$	44.1/45.1	41.8/41.7	400
${\text{TE}}_{00}$/${\text{TE}}_{03}$	45.1/43.6	43.4/39.4	400
${\text{TE}}_{00}$/${\text{TE}}_{03}$	48.8/45.0	42.9/43.1	300

*Note:* Values are rounded to one decimal place.

Figure 6 presents the experimental characterization of the ${\text{TE}}_{00}$–${\text{TE}}_{03}$ BiMC fabricated on the 300‐nm SiN platform at INRS. Figure 6a shows the optimized device geometry with a footprint of 32 μm × 7 μm. Similar to the ANT platform, the black regions correspond to the SiN core. The SEM image shown in Figure 6b confirms the accurate fabrication of the inverse‐designed structure. Several sub‐MFS features with dimensions close to 100 nm, highlighted using green circles, are successfully reproduced despite being smaller than the nominal 120‐nm minimum feature size. These results demonstrate the high fabrication reliability achieved by the INRS process.

**FIGURE 6:**
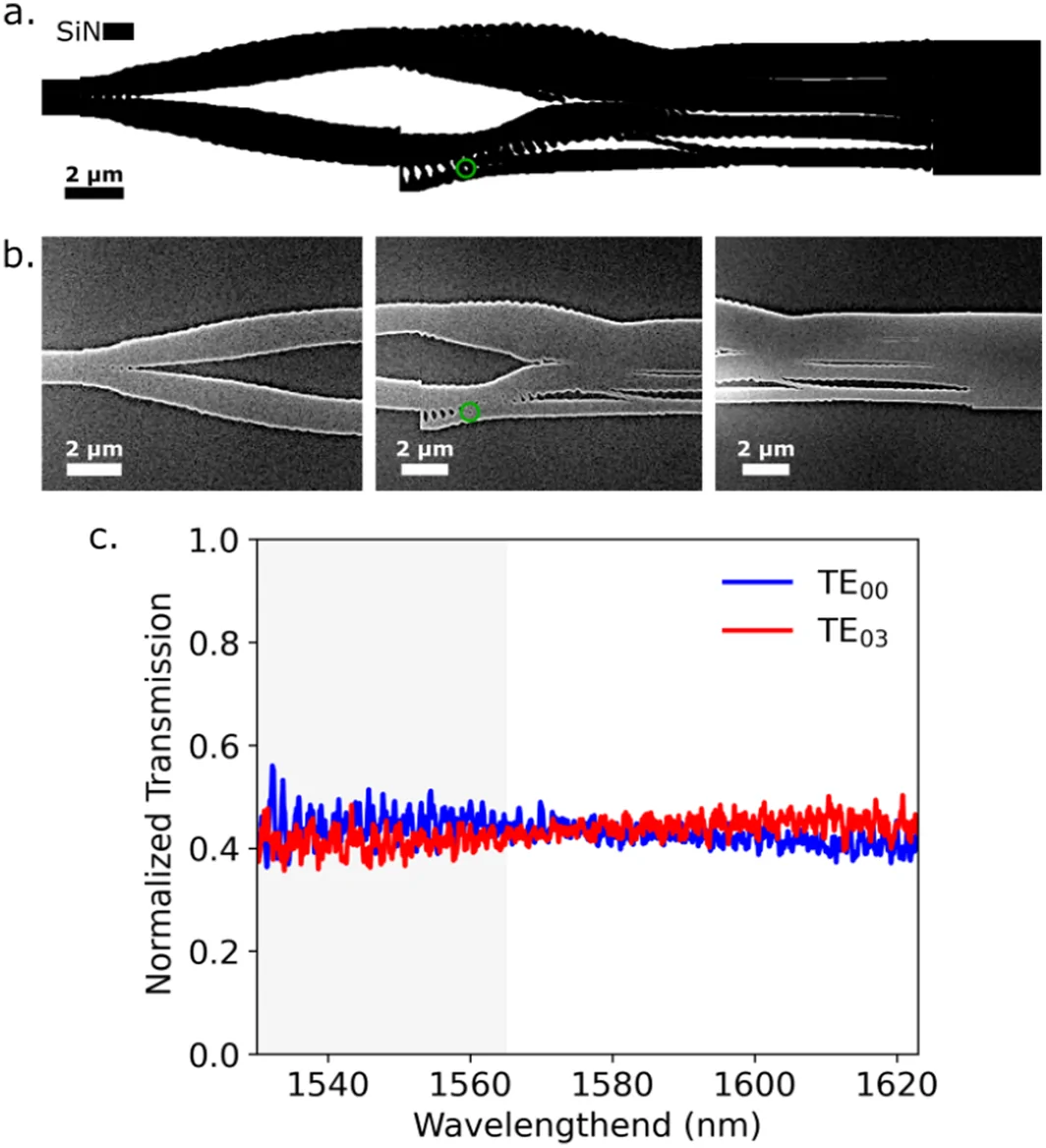
Fabricated inverse‐designed BiMC and experimental characterization on the 300‐nm SiN platform. (a) Geometry of the ${\text{TE}}_{00}$–${\text{TE}}_{03}$ BiMC with a footprint of 32 μm × 7 μm. (b) SEM image of the fabricated device obtained using the INRS fabrication process. (c) Measured transmission spectra and mode‐conversion efficiency across the C and L bands.

Figure 6c presents the measured transmission spectra of the device. Across the C band, the average modal transmission is 44.4% for the ${\text{TE}}_{00}$ mode and 41.1% for the ${\text{TE}}_{03}$ mode, whereas the corresponding L‐band averages are 42.1% and 44.2%, respectively. At 1550 nm, the measured modal power distribution is 42.5%/41.8%. The best balanced split is obtained at 1590.03 nm, where both modes reach 43.0% transmission. The worst‐case split within the C band occurs at 1532.32 nm, corresponding to 54.7%/37.4%. As observed for the 400‐nm platform, spectral oscillations are present due to the grating‐coupler normalization procedure and residual Fabry–Pérot resonances associated with the subwavelength features of the device. The maximum oscillation amplitude reaches approximately 19%. Nevertheless, the experimental measurements remain in good agreement with the simulations, demonstrating the versatility of the inverse‐design methodology and its ability to maintain balanced modal excitation across different SiN platform thicknesses.

The experimental results indicate a gradual increase in insertion loss and modal imbalance toward the L band, consistent with the C band‐centered optimization strategy. However, for the 300‐nm platform, the best modal power balance is obtained near 1590.03 nm within the L band.

Among the devices fabricated on the 400‐nm platform, the ${\text{TE}}_{00}$–${\text{TE}}_{03}$ BiMC exhibits the highest insertion loss. Across the full wavelength range, the insertion loss of the ${\text{TE}}_{00}$ mode remains below 1.8 dB, whereas the ${\text{TE}}_{03}$ mode remains below 4.5 dB. In contrast, the 300‐nm platform demonstrates lower overall losses, even outside the optimized wavelength range, with worst‐case insertion losses of approximately 1.8 dB for the ${\text{TE}}_{00}$ mode and 1.5 dB for the ${\text{TE}}_{03}$ mode.

To experimentally validate the impact of balanced modal excitation on interferometric visibility, a complete bimodal interferometer was fabricated on the 300‐nm SiN platform, as illustrated in Figure 7a. The device integrates two identical inverse‐designed BiMCs arranged back to back and connected through a multimode waveguide with a length of L=15mm. When the interferometer is used as a sensor, the multimode waveguide will act as the sensing region. Figure 7b presents the measured transmission spectrum of the interferometric device. Sharp and highly stable interference fringes are observed across both the C and L bands, with an average free spectral range (FSR) of approximately 1.92 nm. The high spectral contrast confirms efficient excitation and recombination of the ${\text{TE}}_{00}$ and ${\text{TE}}_{03}$ modes.

**FIGURE 7:**
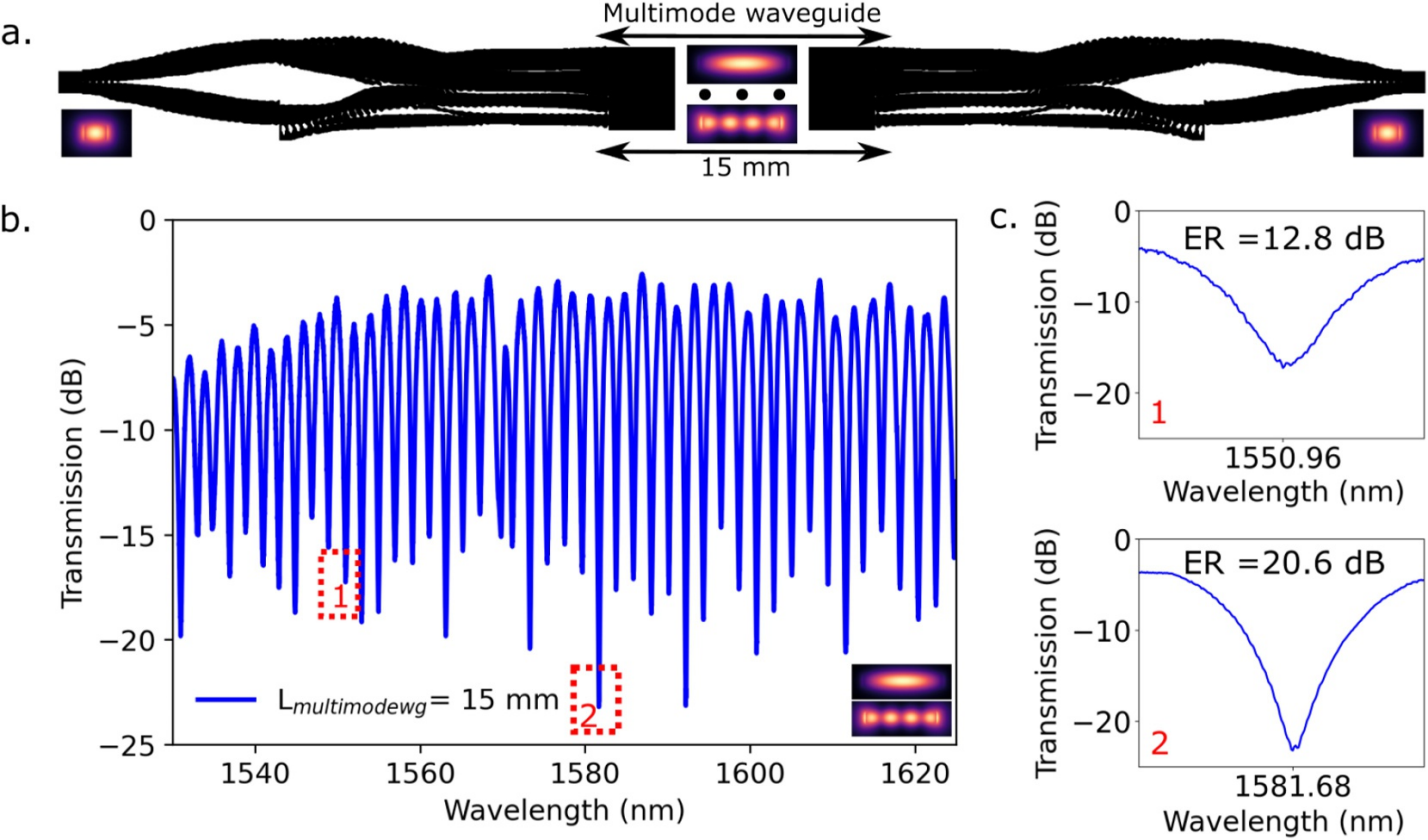
Experimental demonstration of a bimodal interferometer on the 300‐nm SiN platform. (a) Schematic of the interferometer incorporating two inverse‐designed BiMCs connected by a 15‐mm multimode waveguide supporting the ${\text{TE}}_{00}$ and ${\text{TE}}_{03}$ modes. (b) Measured interference spectrum exhibiting stable high‐visibility fringes across the C and L bands. (c) Magnified views of resonances at 1581.68 and 1550.96 nm, showing extinction ratios of 20.6 and 12.8 dB, respectively.

Figure 7c highlights resonances located at 1581.68 and 1550.96 nm. At 1581.68 nm, the device exhibits an experimental extinction ratio (ER) of 20.6 dB, which corresponds to a fringe visibility of approximately 0.98 and represents, to the best of our knowledge, one of the highest experimentally reported values for a SiN‐based bimodal interferometer. The corresponding BiMC exhibits a modal power distribution of 43.8%/45.5%. At 1550.96 nm, the measured ER is 12.8 dB, corresponding to a fringe visibility of approximately 0.90 and a modal power distribution of 44.3%/37.2% in the associated BiMC.

These results confirm that the balanced modal excitation achieved by the inverse‐designed BiMCs is effectively preserved within the complete interferometer, enabling stable high‐visibility interference even for higher‐order mode combinations. Furthermore, the measured transmission level indicates that the total insertion loss of the interferometer, including the 15 mm multimode waveguide, remains approximately 5 dB over the measured wavelength range.

## 4 Discussion and Conclusion

SiN is attractive for integrated biosensing because of its compatibility with CMOS fabrication processes, low propagation loss, and broad optical transparency extending from the visible to the near‐infrared. However, the realization of compact interferometric sensors with SiN requires efficient on‐chip modal control components capable of exciting higher‐order modes while maintaining balanced modal power distributions. This requirement is particularly critical in bimodal interferometric sensors, where the extinction ratio and fringe visibility strongly depend on the optical power balance between the interfering modes. In practice, compact SiN mode converters often suffer from reduced higher‐order mode excitation efficiency, increased modal crosstalk, or limited flexibility when targeting different mode combinations.

In this work, these limitations are addressed through the experimental demonstration of inverse‐designed bimodal mode converters capable of exciting multiple mode pairs, including the challenging ${\text{TE}}_{00}$–${\text{TE}}_{03}$ configuration. The proposed devices achieve highly balanced modal power distributions within ultra‐compact footprints as small as 32 μm × 7 μm. On the 300‐nm SiN platform, the experimentally measured ${\text{TE}}_{00}$–${\text{TE}}_{03}$ BiMC achieves average modal transmissions of 44.4% and 41.1% across the C band for the ${\text{TE}}_{00}$ and ${\text{TE}}_{03}$ modes, respectively.

Most importantly, experimental validation of a complete interferometer demonstrates stable high‐visibility modal interference enabled by the proposed BiMCs. The fabricated interferometric device exhibits an extinction ratio of 20.6 dB, corresponding to a fringe visibility of approximately 0.98. To the best of our knowledge, this represents one of the highest experimentally reported fringe visibilities for a SiN‐based bimodal interferometric device. These results confirm that the balanced modal excitation generated by the inverse‐designed BiMCs is effectively preserved over a 15‐mm multimode waveguide, enabling low‐loss and high‐contrast interference even for higher‐order mode combinations.

Existing modal‐excitation strategies for bimodal interferometers often rely on horizontally shifted junctions [8, 39, 47], directional couplers [5], or periodic structures such as subwavelength gratings [40, 41] and long‐period gratings [42]. Although these approaches can provide efficient mode conversion, balanced excitation of higher‐order modes within compact footprints remains difficult, particularly on low‐index‐contrast SiN platforms. In addition, many previously reported devices prioritized other performance metrics and did not characterize experimentally the modal power balance required for high‐visibility interferometric sensing. For example, a SWG‐based mode converter implemented on a silicon‐on‐insulator platform demonstrated a ${\text{TE}}_{00}$/${\text{TE}}_{02}$ modal power distribution of 45%/48% within a 9‐μm footprint [41]. However, no complete interferometer based on this approach has been experimentally demonstrated. Polymer‐based implementations have either remained theoretical, as in a long‐period‐grating design reporting a 42%/43% modal power split [42], or experimentally exhibited strongly unbalanced modal excitation, as in the 38.5%/6.5% ${\text{TE}}_{00}$/${\text{TE}}_{02}$ distribution reported in ref. [43]. On SiN platforms, shifted‐junction structures demonstrated for the ${\text{TE}}_{00}$/${\text{TE}}_{01}$ mode pair [47] are compact and fabrication‐friendly, but they remain limited to lower‐order modes and exhibit significant excitation of undesired modes, approaching 10%. Furthermore, although exact measured power ratios are not explicitly detailed in the text, the experimental data provided reveal a distinct modal imbalance, with the ${\text{TE}}_{00}$ power exceeding 50% and the ${\text{TE}}_{01}$ power remaining below 45%.

A comprehensive comparison with previously reported bimodal excitation architectures is provided in Table 3. Compared to these approaches, the proposed inverse‐designed BiMCs provide three key advantages: compact footprint, flexible excitation of multiple supported mode pairs, and experimentally demonstrated balanced modal excitation within a complete interferometer. The ability to efficiently excite the ${\text{TE}}_{03}$ mode while maintaining near‐balanced power splitting is particularly important since higher‐order modes exhibit larger evanescent fields and therefore stronger interaction with the surrounding analyte, which is predicted to improve sensing sensitivity. In addition to compactness and modal balance, the demonstrated fabrication robustness across two independent SiN fabrication processes further highlights the practicality of the proposed inverse‐design strategy. The successful fabrication of several sub‐MFS features together with the experimentally observed agreement between simulation and measurement indicate that inverse‐designed free‐form structures can be reliably implemented using SiN nanofabrication processes.

**TABLE 3:** Comparison of the proposed SiN BiMC with previously reported bimodal excitation architectures.

Ref.	Design	Material	Modes	Length	Operating band	Modal distribution	ER (dB)	Status
[41]	SWG	Si	${\text{TE}}_{00}$/${\text{TE}}_{02}$	9.3 µm	1550 nm	45/48	NA	Fabricated
[39]	Shifted‐junction	Si	${\text{TE}}_{00}$/${\text{TE}}_{01}$	0	1550 nm	NA	15	Fabricated
[42]	LPG	Polymer	${\text{TE}}_{00}$/${\text{TE}}_{02}$	138.8 µm	633 nm	42/43	43	Simulated
[43]	Shifted‐junction	Polymer	${\text{TE}}_{00}$/${\text{TE}}_{02}$	0	C band	38.5/6.5	5.7	Fabricated
[8]	Shifted‐junction	Polymer	${\text{TE}}_{00}$/${\text{TE}}_{01}$	0	890–910 nm	NA	18	Fabricated
[5]	Directional coupler	SiN	${\text{TE}}_{00}$/${\text{TE}}_{04}$	23.9 µm	633 nm	47.9/47.9	NA	Simulated
[47]	Shifted‐junction	SiN	${\text{TE}}_{00}$/${\text{TE}}_{01}$	0	C band	50/45	NA	Fabricated
This work	Inverse‐designed	SiN	${\text{TE}}_{00}$/${\text{TE}}_{03}$	32 µm	C + L band	42.9/43.1	20.6	Fabricated

Overall, inverse‐designed BiMCs with footprints as small as 26 μm × 7 μm for the ${\text{TE}}_{00}$–${\text{TE}}_{01}$ mode pair and 32 μm × 7 μm for the ${\text{TE}}_{00}$–${\text{TE}}_{02}$ and ${\text{TE}}_{00}$–${\text{TE}}_{03}$ mode pairs were experimentally demonstrated on both 300 and 400 nm SiN platforms. A complete bimodal interferometer based on the ${\text{TE}}_{00}$–${\text{TE}}_{03}$ BiMCs achieved an extinction ratio of 20.6 dB, corresponding to a fringe visibility of approximately 0.98. These results establish inverse‐designed BiMCs as compact, fabrication‐robust, and highly versatile building blocks for next‐generation integrated bimodal interferometers targeting sensing applications.

## Author Contributions

**Narges Dalvand:** investigation, writing – original draft, methodology, validation, visualization, writing – review and editing, software, formal analysis, project administration, data curation, conceptualization. **Julian L. Pita Ruiz:** writing – review and editing, software, validation, conceptualization, methodology, investigation, formal analysis, visualization. **Boris Le Drogoff:** writing – review and editing, investigation, project administration, validation, data curation. **Mohamed Chaker:** funding acquisition, resources, supervision, writing – review and editing, project administration. **Michaël Ménard:** conceptualization, supervision, project administration, funding acquisition, writing – review and editing, resources, formal analysis.

## Funding

This work was supported by the Natural Sciences and Engineering Research Council of Canada (NSERC), PRIMA Québec, and AEPONYX Inc.

## Conflicts of Interest

The authors declare no conflicts of interest.

## Supporting information


Supporting Information S1

## Data Availability

Data generated and analyzed during this research can be obtained from the corresponding author upon reasonable request.
