# Supplementary material for: Inverse‐Designed Silicon Nitride Bimodal Mode Converter for Interferometric Sensors
Source: Nanophotonics. 2026 Jul 25;15(14):e70229. doi: 10.1002/nap2.70229 (PMC13642527; doi:10.1002/nap2.70229)
Supplement: Supplementary file 1 [file NAP2-15-e70229-s001.pdf]

Supplementary Information: Inverse-Designed Silicon Nitride Arbitrary Bimodal Mode Converter for Interferometric Sensors

Narges Dalvand,† Julian L. Pita Ruiz,† Boris Le Drogoff,‡ Mohamed Chaker,‡ and Michaël Ménard*,†

†*Department of Electrical Engineering, École de Technologie Supérieure (ÉTS), Montreal, QC H3C 1K3, Canada*

‡*Institut national de la recherche scientifique – Énergie, Matériaux Télécommunications (INRS-EMT), Varennes, Canada*

E-mail: michael.menard@etsmtl.ca

Supplementary Note 1: Inverse Design Process

Supplementary Figure S1 illustrates the inverse design framework. Initially, the optimization was performed using a single design region located between the input single-mode waveguide and the output multimode waveguide, as shown in Figure S1(a). This region was initialized with a uniform SiN or silicon dioxide material distribution. The figure of merit (FOM) was defined to maximize the coupling efficiency to the two target modes by minimizing $\text{FOM} = (1 - \eta_1) + (1 - \eta_2)$, where η_1 and η_2 represent the coupling efficiencies of the two target modes at the output multimode waveguide. However, with this configuration the optimization failed to converge, as shown in Figure S1(d).

To address this issue, the optimization was divided into two stages: the design of a beam splitter and the design of a mode converter region, as shown in Figure S1(b). The mode converter was first optimized using two input waveguides, each targeting a different mode in the output multimode waveguide, as illustrated in Figure S1(c). For instance, in the structure to the left in Figure S1(c), the upper input single-mode waveguide was configured to excite the TE_{00} mode in the output multimode waveguide. In the structure to the right, the lower input single-mode waveguide was configured to convert the input mode into an higher-order mode of the output multimode waveguide. To further promote convergence, two S-bend paths were used as the initial geometry, as also shown in Figures S2(b) and S2(c). Because the optimized mode converter requires two independent inputs, an additional optimization step was performed to design a compact beam splitter that divides the optical signal from the input single-mode waveguide.

Figure S1(d) shows the optimization trajectory obtained with a uniform initial condition, where the FOM does not converge and the fundamental mode remains nearly constant throughout the iterations. In contrast, Figure S1(e) shows the optimization trajectory obtained using the initialization with S-bends, which converges successfully. For both convergence trajectories, three preliminary geometries are shown. Although the TE_{00}–TE_{01} design process is presented here as an example, a similar behavior was observed for the other mode

pairs. The number of iterations required for each component varied depending on the design, ranging from 96 iterations for the beam splitter to 152 iterations for the TE_{00}–TE_{03} mode converter.

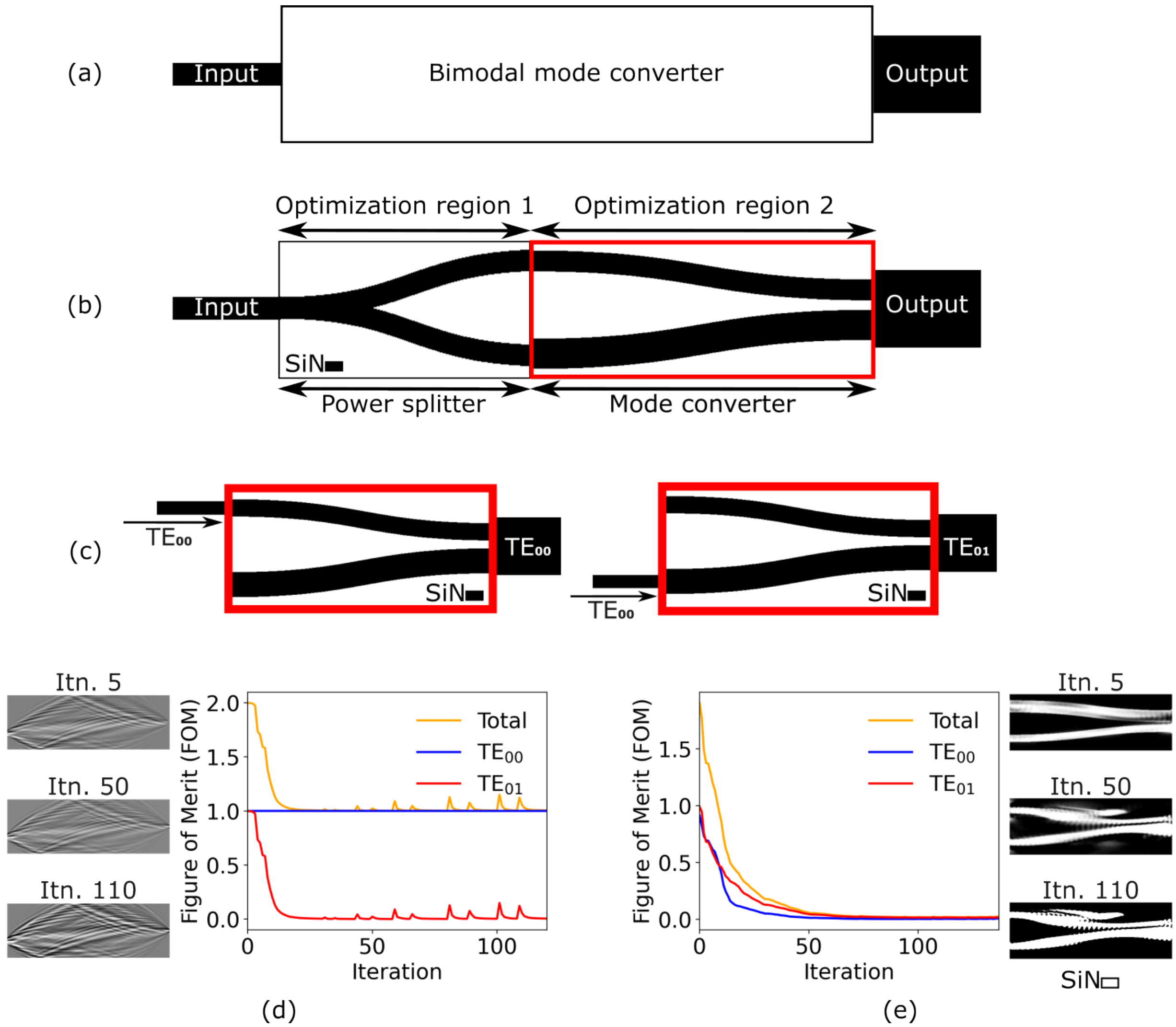

Figure S1: (a) Definition of the optimization region without any structured initial condition. (b) Division of the optimization region into a beam splitter and a mode converter, using SiN S-bends as the initial condition. (c) Schematic showing the two separate paths in the mode converter that guide the fundamental and higher-order modes. (d) Optimization trajectories with no specific initial condition and with the structured initial condition, respectively. As an example, the layout for BiMC with the TE_{00}–TE_{01} modes is shown. The structures obtained after iterations 5, 50, and 110 are included to better illustrate the optimization process.

After the optimization stage, the geometries of the individual components were extracted

and evaluated together in a separate three-dimensional FDTD verification simulation using an ultra-fine mesh. The coupling efficiencies obtained from the optimization and verification simulations showed good agreement.

Supplementary Note 2: Simulation of Fabrication Variations

Fabrication-induced dimensional variations were studied to evaluate the robustness of the components. To emulate these tolerances, dimensional variations were modeled using global erosion and dilation operations applied to the complete device geometry. Lateral undersizing and oversizing of approximately $\pm 10\,\text{nm}$ and $\pm 20\,\text{nm}$ were applied to the nominal device geometry. To achieve high robustness in our devices, we carefully tuned the thresholding-sharpness parameters during the optimization process, combined with a controlled extraction-threshold strategy for the final non-fully-binary geometry.

Figure S2 shows the simulation results for the BiMCs on 400 nm-thick and 300 nm-thick SiN platforms. The analysis was performed while keeping the device thickness constant. The response of the TE_{00}–TE_{01} device on the 400 nm platform, illustrated in the top row of Figure S2, demonstrates high stability under a $\pm 10\,\text{nm}$ variation. The power splitting remains highly balanced across the C-band, yielding average transmissions of 47.1% for TE_{00} and 45.3% for TE_{01} under lateral undersizing, and 44.1% for TE_{00} and 47.7% for TE_{01} under lateral oversizing. When the variation increases to $\pm 20\,\text{nm}$, the device exhibits its highest sensitivity under lateral undersizing; in this worst-case scenario, the transmission balance deviates to 55.6%/35.7% at the critical wavelength of 1565 nm. Conversely, under 20 nm oversizing, the transmission remains robust, with average values of 43.6%/46.7% for TE_{00}/TE_{01} across the C-band.

For the TE_{00}–TE_{02} device on the 400 nm platform, shown in the second row of Figure S2, the device displays stable performance under $\pm 10\,\text{nm}$ variations. Under the $+10\,\text{nm}$ varia-

tion, the average power ratio settles at 47.8%/43.1% across the C-band. Under the -10 nm variation, a slightly larger separation in power occurs, with the average power splitting across the C-band reaching 41.8%/48.3%. Increasing the variation to ±20 nm further broadens the power gap; however, the best power splitting for the TE_{00}–TE_{02} device under undersizing occurs at the edge of the C-band (1565 nm), with a value of 49.5%/39.6%, while the best-case performance under oversizing is observed at 1530 nm, with a value of 37.6%/49.3%.

The TE_{00}–TE_{03} device on the 400 nm platform (third row) demonstrates even greater robustness against lateral variations compared to the previously described devices. For ±10 nm variations, the transmission responses for both modes closely track each other. Under severe ±20 nm variations, the largest performance degradation occurs for 20 nm oversizing, pushing the power ratio to a worst-case value of 46.8%/34.3% at 1530 nm, while the average power ratio remains at 45.2%/37.2% across the C-band.

Finally, the bottom row of Figure S2 depicts the TE_{00}–TE_{03} device implemented on the thinner 300 nm SiN platform. A ±10 nm variation results in a well-balanced average transmission of 48.2% for TE_{00} and 45.2% for TE_{03} under undersizing variations, and 46.6% for TE_{00} and 44.6% for TE_{03} under oversizing variations. For ±20 nm variations, this configuration proves to be more tolerant to undersizing than to oversizing within the C-band, with the worst-case imbalance recorded at 1530 nm for oversizing variations, where the power-splitting ratio reaches 41.9%/33.8%.

Overall, across all simulated devices, the modal excitation remains within acceptable operational limits, confirming the effectiveness of the inverse design approach. The average transmission for each mode pair on both SiN platform thicknesses is also summarized in Table S1 to provide a more intuitive representation of the device efficiency.

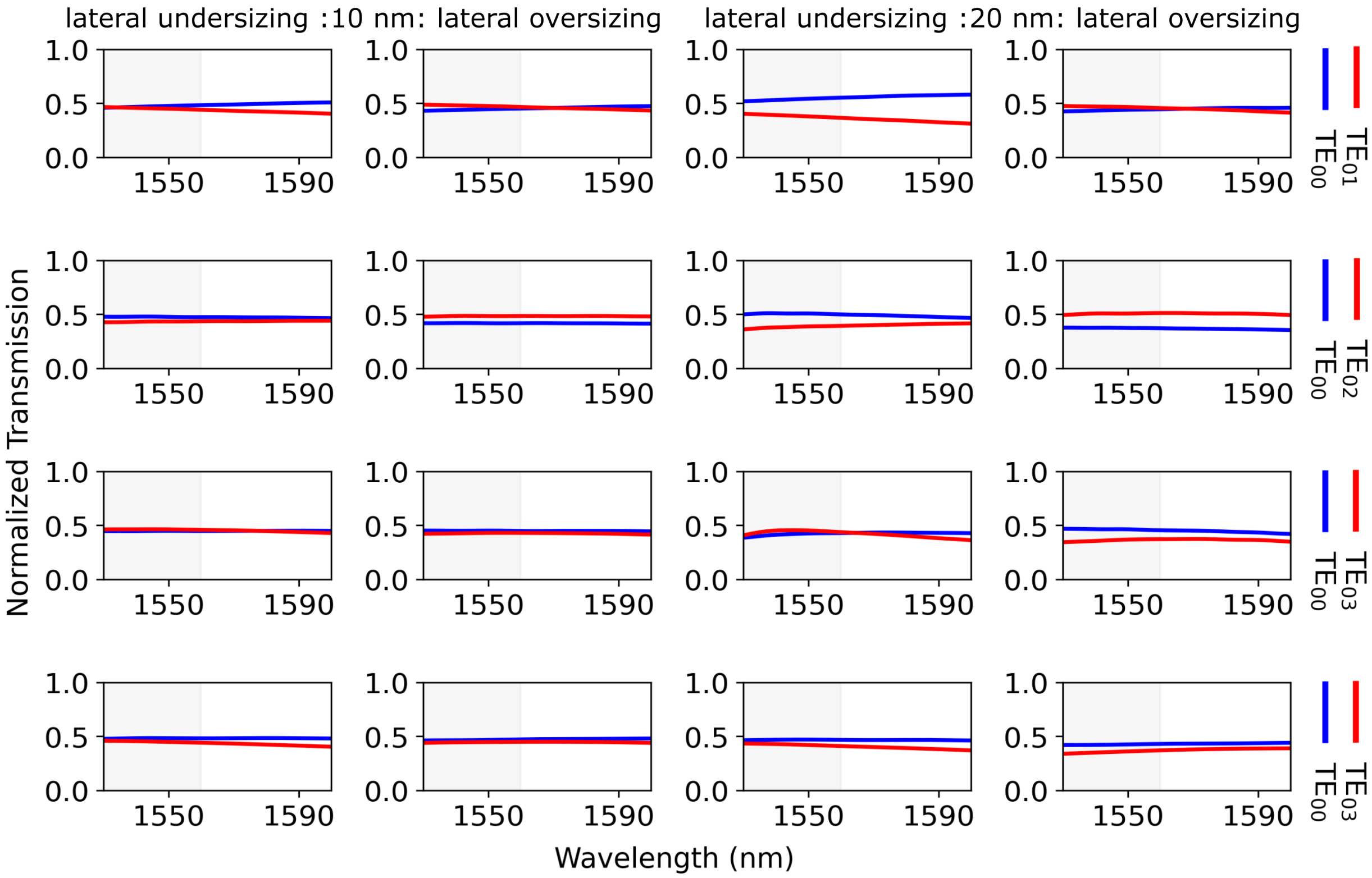

Figure S2: Performance of each design under 10 nm and 20 nm lateral undersizing and oversizing for both 400 nm and 300 nm thick SiN platforms. The first three rows show the results for TE_{00}–TE_{01}, TE_{00}–TE_{02}, and TE_{00}–TE_{03}, respectively, on the 400 nm thick platform, while the last row shows the results for the TE_{00}–TE_{03} pair on the 300 nm thick platform.

Table S1: Average transmission for each mode pair under 10 nm and 20 nm of lateral undersizing (under) and lateral oversizing (over) in the 400 nm and 300 nm SiN platform. Values are rounded to one decimal place.

	Lateral undersizing (%/%)		Lateral oversizing (%/%)	
	10 nm	20 nm	10 nm	20 nm
TE_{00}/TE_{01} (400 nm)	48.5/43.5	55.4/35.8	45.3/46.1	44.5/44.9
TE_{00}/TE_{02} (400 nm)	47.3/43.5	49.3/39.5	41.7/48.3	36.8/50.6
TE_{00}/TE_{03} (400 nm)	44.8/45.2	42.4/42.0	44.8/42.5	45.0/36.4
TE_{00}/TE_{03} (300 nm)	48.2/43.6	46.7/40.5	47.1/44.6	42.9/37.0

Supplementary Note 3: Prefabrication Corrections

To improve the performance of the devices, we used a deep learning model from PreFab Photonics,[1] trained on the Applied Nano Tools (ANT) silicon nitride fabrication process. The model was trained using SEM images and the corresponding layout patterns to capture how the ANT silicon nitride process modifies the intended design during fabrication. It then uses this learned behavior to pre-adjust the layout prior to fabrication, helping compensate for lateral deviations such as rounded corners.

Figure S3(a) and S3(b) show the original and refined layouts, respectively. Figure S3(c) presents zoomed-in views of the regions highlighted in (a) and (b). Small holes were added or removed, and the edges in the refined layout are sharper than the smoother edges of the original design. These modifications slightly improved fabrication fidelity and consequently enhanced device performance, resulting in experimental results that more closely match the simulations. Figure S3(d) shows a zoomed-in SEM image of the fabricated device, where the red rectangle outlines the specific region highlighted in the layouts in Figure S3(b) and (c). As observed in the SEM image, the fabrication-aware inverse-design methodology ensures that the geometric features of the fabricated devices closely match the originally intended layout.

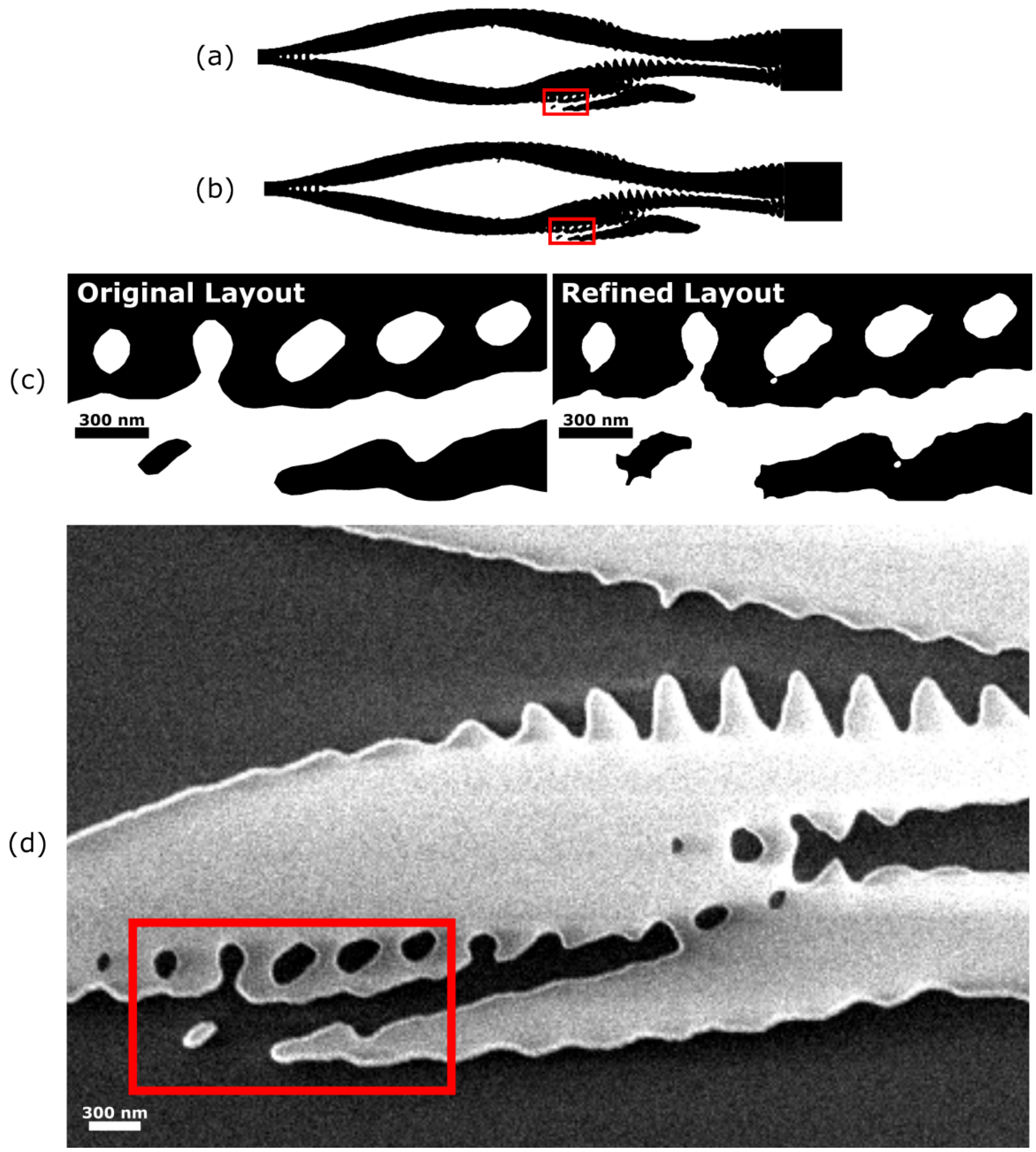

Figure S3: Comparison between the original and refined layouts for BiMC: TE_{00}–TE_{01}. (a) Original layout, (b) refined layout, and (c) zoomed-in view of the same region showing more details. In (c), the left plot shows the original layout, and the right plot shows the corrected layout. (d) Zoomed-in SEM images of the BiMC fabricated on the 400 nm SiN platform. The red rectangle indicates the region highlighted in (a) and (b). The small holes confirm the successful fabrication of the 120 nm minimum feature size.

References

(1) PreFab-Photonics PreFab: Artificial nanofabrication of integrated photonic circuits using deep learning. `https://github.com/PreFab-Photonics/PreFab`, Accessed: 2025-03-18.
